# Survival analysis of older adults with dementia: predicting factors after unplanned hospitalization in Maharaj Nakorn Chiang Mai Hospital

**DOI:** 10.1186/s12877-023-04558-x

**Published:** 2024-01-03

**Authors:** Thanachat Yotruangsri, Phichayut Phinyo, Nida Buawangpong, Nopakoon Nantsupawat, Chaisiri Angkurawaranon, Kanokporn Pinyopornpanish

**Affiliations:** 1 Department of Family Medicine, Faculty of Medicine, Chiang Mai University, 110 Inthawarorot Rd., Sriphum, Muang, Chiang Mai, 50200 Thailand; 2 Center for Clinical Epidemiology and Clinical Statistics, Faculty of Medicine, Chiang Mai University, Chiang Mai, 50200 Thailand; 3 Musculoskeletal Science and Translational Research (MSTR), Chiang Mai University, Chiang Mai, 50200 Thailand; 4 Global Health and Chronic Conditions Research Group, Chiang Mai University, Chiang Mai, 50200 Thailand

**Keywords:** Dementia, Alzheimer’s Disease, Hospitalization, Survival time, Mortality

## Abstract

**Background:**

Hospitalization in individuals with dementia can be associated with negative and unintended outcomes. Research indicates that people with dementia experience more hospital admissions in comparison to individuals without dementia. This study aims to assess the survival time of individuals with dementia who experience unplanned hospitalization and examine the factors that are associated with mortality in this population.

**Methods:**

This retrospective cohort study was conducted using data from older adults with dementia who survived unplanned hospitalizations at Maharaj Nakorn Chiang Mai Hospital between January 1, 2009, and December 31, 2016. The association between factors and mortality were analyzed using a multivariable Cox proportional hazards model.

**Results:**

One hundred and eighty-one cases were included. The mean age of the study population was 80.07 (SD 7.49) years, and the majority were female (56.91%). The median survival time of the studied cohort was 3.06 years (95% CI 3.14–3.60). The multivariable analysis revealed that older age (aHR = 1.02, 95% CI 1.00-1.05), a diagnosis of mixed-type dementia (aHR = 3.45, 95% CI 1.17–10.14), higher Charlson comorbidity index score (aHR = 1.19, 95% CI 1.04–1.36), higher serum creatinine level (aHR = 1.35, 95% CI 1.10–1.66), insertion of endotracheal tube (aHR = 1.95, 95% CI 1.07–3.54), and readmission within 30 days (aHR = 1.88, 95% CI 1.18–2.98) were associated with an increased risk of mortality.

**Conclusions:**

We identified several notable predictors of mortality. Healthcare providers can use the findings of this study to identify patients who may be at higher risk of mortality and develop targeted interventions which may improve patient outcomes.

**Supplementary Information:**

The online version contains supplementary material available at 10.1186/s12877-023-04558-x.

## Background

Dementia is a prevalent and disabling condition that significantly impairs the social functioning of individuals [1], with advanced age being a major risk factor for its development [2]. The worldwide prevalence of dementia among older adults is projected to increase substantially in the foreseeable future [3].

A systematic review, utilizing hospital administrative databases to compare the outcomes of people with dementia to elderly individuals without dementia, reported that those with dementia had a higher overall readmission rate [4]. Research indicates that people with dementia experience 1.4 to 4 times more hospital admissions compared to individuals without dementia [5]. After the diagnosis of dementia, the rate and length of unplanned hospitalizations are typically low and short. However, the admission rate and length tend to increase as individuals approach the end of life [6]. Additionally, they were found to be at an increased risk of developing various complications, including urinary tract infections, pressure ulcers, pneumonia, delirium, dehydration, and electrolyte imbalance [4]. Hospitalization in individuals with dementia is linked to negative and unintended outcomes, including emotional distress, a decline in functioning and cognition, and a significant financial burden.

While most studies have focused on the overall mortality rate of people with dementia after diagnosis [7], there is limited information available about the mortality rate specifically after hospitalization. When compared to non-dementia patients, people with dementia had significantly higher mortality rates. One study found that nearly half of the cohort with dementia had died 12 months after unplanned acute hospitalization [8]. The in-hospital death rate for dementia patients may be about one-fifth of the cases admitted [9]. It is highly likely that caring for individuals with dementia who survived after unplanned admission would require strong support to stay at home. Families may face challenging times after hospitalization, requiring additional support and resources.

Factors associated with adverse outcomes in dementia, when compared to individuals without dementia, include the severity of dementia, the number of types of medication, and the deficit in activity of daily living [10]. In addition, advanced age, hospitalization for myocardial infarction, high serum sodium levels, lower Barthel index, and the incidence of complications during hospitalization have been identified as predictors for higher mortality in people with dementia [11]. Studies in Thailand have identified mortality risk factors in people with dementia [12, 13]. However, specific factors associated with mortality after surviving unplanned hospitalization have not been reported in the available studies.

As unplanned hospital admission may indicate poor or deteriorating health, which is one of the indications for initiation of palliative care according to the Supportive and Palliative Care Indicators Tool (SPICT) [14], it is useful to assess the survival time and factors associated with prognosis. Therefore, our research aims to achieve two main objectives. Firstly, we seek to assess the survival time of individuals with dementia who have survived unplanned hospitalizations. By understanding the length of survival in this specific population, we can gain valuable insights into their long-term prognosis. Secondly, we aim to examine the factors that are associated with mortality in individuals with dementia who experience unplanned hospitalizations. By identifying these factors, we can provide healthcare providers with important information to predict risks, set realistic expectations, communicate with caregivers, and develop tailored care plans for this vulnerable population to improve their quality of life.

## Methods

### Study design and setting

This retrospective cohort study was conducted at Maharaj Nakorn Chiang Mai Hospital, a university-affiliated, tertiary care center in Northern Thailand.

### Study population

We included the data from the medical records of older adults (aged 60 years old and older) with dementia who survived unplanned hospitalizations at Maharaj Nakorn Chiang Mai Hospital. The diagnosis of dementia was determined based on the International Classification of Diseases, 10th Revision (ICD-10) codes recorded during outpatient clinic visits prior to the time of admission. The specific ICD-10 codes used for the diagnosis of dementia included F00-F03 and G30-G32 (Supplementary Table 1). Patients who were diagnosed at the time of hospitalization or those who died during their hospital stay were excluded from the study. Unplanned hospital admissions were defined as hospital admissions or readmissions involving an overnight stay that had not been prearranged or scheduled and were not elective [15].

We applied the *power cox* command for sample-size calculation for survival analyses using Cox proportional hazards models, taking a mortality proportion from a previous study in the literature (48.3%) [8]. With a sample size of 116 cases, the study would have the capacity to detect hazard ratios for each variable at 2.5, achieving a statistical power of 80%. We aimed to collect all cases that met the inclusion criteria to achieve this figure.

### Data collection

The electronic medical records of all relevant patients who were admitted between January 1, 2009, and December 31, 2016, were reviewed. Data on demographic and clinical characteristics were collected, including age, gender, health insurance, admission details (admission date, number of admissions, readmission, type of admission, discharge status, length of stay), primary diagnosis using International Classification of Diseases, Tenth Revision (ICD-10) codes, procedures during hospitalization using International Classification of Diseases, Ninth Revision (ICD-9) codes, department of hospitalization, laboratory results (hemoglobin, hematocrit, white blood cell count, serum creatinine, albumin, sodium, potassium), medication, presence of pressure ulcers, dementia status (type of dementia, Thai Mental State Examination (TMSE) score, Behavioral and Psychological Symptoms of Dementia (BPSD)), underlying conditions using the Charlson Comorbidity Index (CCI) score, level of consciousness, and survival status. For laboratory results, data were collected only from the first laboratory test conducted during admission. All-cause death status and death date were obtained from the Thai civil registration system database. The data retrieving process was conducted by one researcher (TY), and only data from the first admission was included for patients who had been hospitalized more than once during the recruitment period.

### Statistical analysis

All statistical analyses were performed using STATA statistical software version 16. Descriptive statistics were used to summarize the data, with continuous data presented as mean and standard deviation for normally distributed data and median and interquartile range for non-normally distributed data. Categorical data are presented as frequency and percentage.

The student t-test was applied for normally distributed continuous data, the Mann-Whitney U test for non-normally distributed continuous data, and the Fisher exact test for categorical data to assess factors associated with death status. Kaplan-Meier survival analysis and the Cox proportional hazards model were used to evaluate the relationship between independent factors and the mortality rate of dementia after hospitalization.

A complete case analysis was conducted. Survival time was determined as the duration between the discharge date and the date of death after discharge or the last date of the investigative period (December 31, 2022). Factors with a significant association from univariable analysis (*p*-value < 0.2) were included in the multivariable analysis. Stepwise regression with backward elimination was used for variable selection to reduce the model (final model). Adjusted Hazard ratios (aHRs) and 95% confidence intervals (95% CI) were estimated, and data was considered to be statistically significant at *p* < 0.05.

## Results

**Fig. 1:**
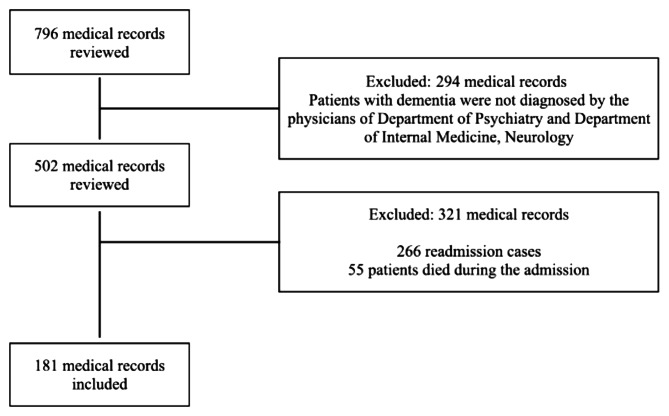
Flow diagram of participant recruitment

A total of 796 medical records were reviewed. Among these, 615 records were excluded from the study for various reasons: 266 records were identified as duplicate cases (readmissions), 55 cases died while in the hospital, and 294 cases were diagnosed at the time of hospitalization. Consequently, 181 cases were included for further analysis. The details are shown in Fig. 1.

The mean age of the study population was 80.07 (SD 7.49) years, and the majority were female (56.91%). The most prevalent types of dementia were Alzheimer’s disease and vascular dementia, and behavioral and psychological symptoms of dementia were present in over half of the participants. Polypharmacy was common (81.22%). Patient characteristics are described in Table 1.

**Table 1:** Patient Characteristics on Admission (N = 181)

Characteristics	Total	Death		*P*-value
		Yes (n = 153)	No (n = 28)	
Age (years), mean ± SD	80.07 ± 7.49	80.41 ± 7.22	78.18 ± 8.76	0.148
Male gender, n (%)	78 (43.09)	71 (46.41)	7 (25.00)	0.035
TMSE score, mean ± SD	15.34 ± 7.58	14.34 ± 7.33	20.43 ± 6.91	< 0.001
Type of dementia, n (%)				0.744
Alzheimer’s disease	60 (33.15)	49 (32.03)	11 (39.29)	
Vascular dementia	66 (36.46)	55 (35.95)	11 (39.29)	
Mixed type	6 (3.31)	6 (3.92)	0 (0.00)	
Other	26 (14.36)	23 (15.03)	3 (10.71)	
Unspecified dementia	23 (12.71)	20 (13.07)	3 (10.71)	
Presence of BPSD	101 (56.11)	87 (57.24)	14 (50.00)	0.478
Chalson Comorbidity Index score, mean ± SD	2.54 ± 1.55	2.60 ± 1.58	2.25 ± 1.29	0.270
General status, n (%)				
Bedridden	41 (22.65)	39 (25.49)	2 (7.14)	0.033
On feeding tube	14 (7.73)	13 (8.5)	1 (3.57)	0.370
On tracheostomy	4 (2.21)	4 (2.61)	0 (0.00)	0.387
Presence of pressure ulcer	18 (9.94)	18 (11.76)	0 (0.00)	0.056
Polypharmacy, n (%)	147 (81.22)	127 (83.01)	20 (71.43)	0.149

**Fig. 2:**
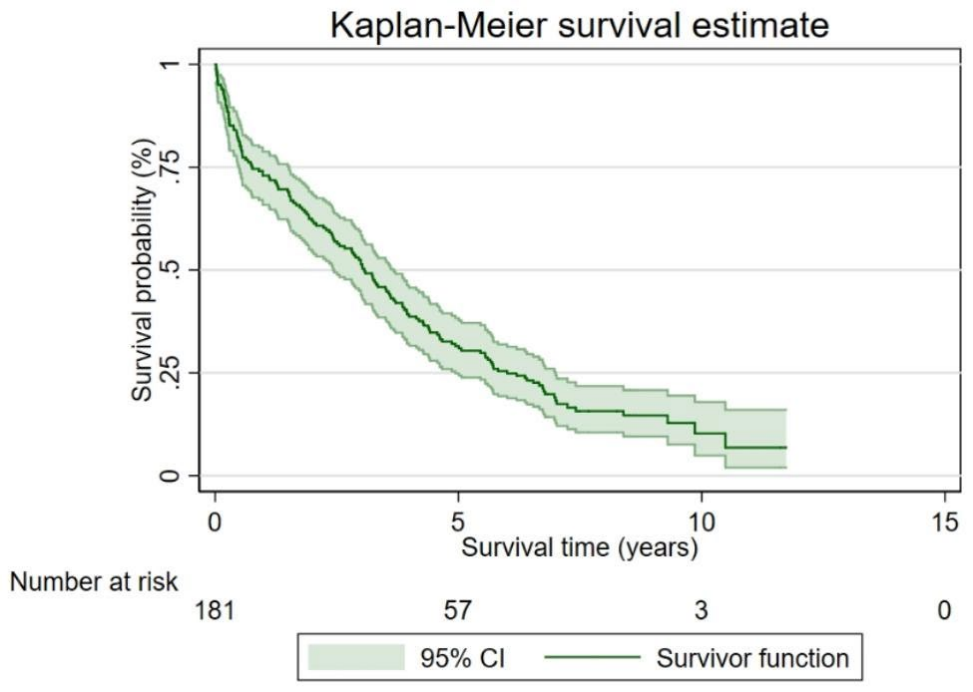
Survival time

The details of primary diagnoses and inpatient departments are outlined in Table 2 and Supplementary Table 2. The breakdown of the primary diagnoses included circulatory (19.89%), respiratory (16.02%), and nervous system-related (11.60%) conditions. Adverse events during hospitalization encompasses infection, respiratory and renal failure, arrhythmias, and delirium. Procedures involved catheterization, oxygen supplementation, and administration of antibiotics. The mean length of stay was 25.46 days, with a readmission rate of 32.61% within 30 days. Information regarding patient status during admission is summarized in Table 3. Figure 2 illustrates the median survival time of the studied cohort to be 3.06 years (95% CI 3.14–3.60). The probabilities of death in this cohort at 1, 5, and 10 years were 27.07%, 68.51%, and 89.76%, respectively.

**Table 2:** Primary Diagnoses and Inpatient Departments

Primary Diagnosis (ICD-10)	n (%)
Diseases of the circulatory system (I00-I99)	36 (19.89)
Diseases of the respiratory system (J00-J99)	29 (16.02)
Diseases of the nervous system (G00-G99)	21 (11.60)
Diseases of the genitourinary system (N00-N99)	19 (10.50)
Injury, poisoning and certain other consequences of external causes (S00-T98)	14 (7.73)
Diseases of the digestive system (K00-K93)	12 (6.63)
Mental and behavioral disorders (F00-F99)	11 (6.08)
Symptoms, signs and abnormal clinical and laboratory findings, not elsewhere classified (R00-R99)	7 (3.87)
Certain infectious and parasitic diseases (A00-B99)	6 (3.31)
Endocrine, nutritional and metabolic diseases (E00-E90)	6 (3.31)
Diseases of the skin and subcutaneous tissue (L00-L99)	6 (3.31)
Neoplasms (C00-D48)	5 (2.76)
Diseases of the blood and blood-forming organs and certain disorders involving the immune mechanism (D50-D89)	4 (2.21)
Diseases of the musculoskeletal system and connective tissue (M00-M99)	3 (1.66)
Diseases of the eye and adnexa (H00-H59)	2 (1.10)
Inpatient Departments	
Internal medicine	134 (74.03)
Surgery	21 (11.60)
Orthopedics	13 (7.18)
Ear Nose Throat	4 (2.21)
Ophthalmology	2 (1.10)
Rehabilitation	1 (0.55)
Obstetrics and gynecology	1 (0.55)
Other departments	5 (2.78)

**Table 3:** Patient Information during Hospitalization and after Discharge (N = 181)

Information	Total	Death		*P*-value
		Yes (n = 153)	No (n = 28)	
Consciousness on admission, n (%)				0.558
Awake	135 (74.59)	112 (73.20)	23 (82.14)	
Drowsiness	45 (24.86)	40 (26.14)	5 (17.86)	
Coma	1 (0.55)	1 (0.65)	0 (0.00)	
Laboratory results, mean ± SD				
Hematocrit (%)	35.33 ± 6.19	35.51 ± 6.11	34.39 ± 6.65	0.382
White blood cells	9154.13 ± 4311.67	9347.68 ± 4439.01	8110.36 ± 3427.74	0.164
Serum albumin (mg/dL)	3.32 ± 0.63	3.26 ± 0.63	3.59 ± 0.55	0.010
Creatinine (mg/dL)	1.42 ± 1.07	1.48 ± 1.13	1.11 ± 0.58	0.091
Serum sodium (mmol/L)	138.92 ± 7.51	139.23 ± 7.76	137.21 ± 5.80	0.193
Serum potassium (mmol/L)	3.95 ± 0.61	3.99 ± 0.61	3.73 ± 0.57	0.035
Adverse events, n (%)				
Delirium	67 (37.22%)	59 (38.82)	8 (28.57)	0.303
Urinary tract infection	37 (20.44%)	34 (22.22)	3 (10.71)	0.208
Acute renal failure	24 (13.26%)	18 (11.76)	6 (21.43)	0.220
Pneumonia	20 (11.11%)	19 (12.50)	1 (3.57)	0.322
Respiratory failure	19 (10.50%)	18 (11.76)	1 (3.57)	0.316
Arrythmia	14 (7.78%)	12 (7.89)	2 (7.14)	1.000
Other infection	10 (5.56%)	9 (5.92)	1 (3.57)	1.000
Myocardial infarction	3 (1.66%)	3 (1.96)	0 (0.00)	1.000
Cerebrovascular event	2 (1.10%)	2 (1.31)	0 (0.00)	1.000
Cardiac arrest	0	0	0	
Procedures, n (%)				
Intravenous antibiotics	113 (62.43%)	96 (62.75)	17 (60.71)	0.835
Oxygen supplementation	95 (52.49%)	80 (52.29)	15 (53.57)	1.000
Insertion of urinary catheter	79 (43.65%)	67 (43.79)	12 (42.86)	1.000
Insertion of nasogastric tube	64 (35.36%)	57 (37.25)	7 (25.00)	0.283
Transfusion of blood and blood components	30 (16.57%)	28 (18.30)	2 (7.14)	0.176
Insertion of endotracheal tube	26 (14.36%)	24 (15.69)	2 (7.14)	0.379
Central venous catheter placement	8 (4.42%)	7 (4.58)	1 (3.57)	1.000
Hemodialysis	3 (1.66%)	3 (1.96)	0 (0.00)	1.000
Infusion of vasopressor agent	1 (0.55%)	1 (0.65)	0 (0.00)	1.000
Cardiopulmonary resuscitation	0	0	0	
Ever transferred to intensive care unit, n (%)	19 (10.56)	18 (11.76)	1 (3.57)	0.316
Length of stay (days), median(IQR)	8 (4–15)	7 (4–15)	8 (3.5–16)	0.677
Number of hospitalizations after first admission, median(IQR)	2.42 (1–3)	1 (1–3)	2 (1-3.5)	0.675
Readmission within 30 days, n (%)	45 (32.61)	40 (34.48)	5 (22.73)	0.330
Follow up time (years), median(IQR)	3.06 (0.76–5.99)	2.42 (0.55–4.28)	7.35 (6.67–8.56)	< 0.001

**Table 4:** Cox Proportional Hazards Models for the Effect of Study Variables on Mortality

	Univariable analysis		Multivariable analysis		Multivariable analysis (reduced model)	
	Crude HR	*p*-value	Adjusted HR	*p*-value	Adjusted HR	*p*-value
Older age	1.02 (1.00-1.04)	0.028	1.04 (1.01–1.08)	0.005	1.02 (1.00-1.05)	0.045
Male gender	1.90 (1.37–2.63)	< 0.001	1.91 (1.11–3.29)	0.019	1.39 (0.91–2.13)	0.122
Type of dementia						
• Alzheimer’s disease	Ref		Ref		Ref	
• Vascular dementia	1.05 (0.71–1.55)	0.805	1.11 (0.59–2.09)	0.740	1.12 (0.67–1.84)	0.662
• Mixed type	2.95 (1.25–6.99)	0.013	3.64 (1.08–12.27)	0.037	3.45 (1.17–10.14)	0.024
• Other	1.33 (0.81–2.19)	0.252	1.44 (0.64–3.24)	0.375	1.52 (0.81–2.84)	0.189
• Unspecified dementia	1.11 (0.66–1.87)	0.688	0.78 (0.29–2.11)	0.629	0.88 (0.39–1.98)	0.772
Higher TMSE score	0.96 (0.94–0.98)	0.002	0.94 (0.91–0.98)	0.003		
Presence of BPSD	1.26 (0.91–1.74)	0.158	0.82 (0.48–1.41)	0.472		
Higher Charlson Comorbidity Index score	1.14 (1.03–1.26)	0.009	1.20 (1.02–1.41)	0.024	1.19 (1.04–1.36)	0.010
Presence of pressure ulcer	2.92 (1.75–4.85)	< 0.001	2.02 (0.69–5.92)	0.198	5.41 (2.71–10.76)	< 0.001
Higher serum albumin	0.55 (0.41–0.74)	< 0.001	0.84 (0.50–1.38)	0.497		
Higher serum creatinine	1.32 (1.14–1.54)	< 0.001	1.26 (0.99–1.59)	0.053	1.35 (1.10–1.66)	0.004
Pneumonia	1.86 (1.15–3.02)	0.011	1.14 (0.48–2.68)	0.759		
Urinary tract infection	1.29 (0.88–1.89)	0.194	0.94 (0.50–1.75)	0.851		
Insertion of endotracheal tube	1.85 (1.19–2.87)	0.006	1.73 (0.68–4.44)	0.248	1.95 (1.07–3.54)	0.027
Insertion of nasogastric tube	1.81 (1.30–2.53)	< 0.001	1.60 (0.85–3.04)	0.147		
Transfusion of blood and blood components	1.93 (1.27–2.92)	0.002	1.51 (0.67-3,39)	0.320		
Readmission within 30 days	1.39 (0.95–2.05)	0.091	1.67 (0.90–3.09)	0.103	1.88 (1.18–2.98)	0.007

Table 4 illustrates the results of the Cox proportional hazards model identifying factors affecting mortality in people with dementia following discharge. The final model of multivariable analysis revealed that older age (aHR = 1.02, 95% CI 1.00-1.05), a diagnosis of mixed-type dementia (aHR = 3.45, 95% CI 1.17–10.14), higher CCI score (aHR = 1.19, 95% CI 1.04–1.36), higher serum creatinine level (aHR = 1.35, 95% CI 1.10–1.66), insertion of endotracheal tube (aHR = 1.95, 95% CI 1.07–3.54), and readmission within 30 days (aHR = 1.88, 95% CI 1.18–2.98) were associated with an increased risk of mortality.

## Discussion

This study reports on the survival time after unplanned hospitalization among Thai older adult patients with dementia. The median overall survival after hospitalization was approximately three years. Older age, higher comorbidity, a higher serum creatinine level, insertion of endotracheal tube. and readmission within 30 days were significantly related to higher mortality.

The demographic characteristics of the study sample indicate some important considerations. First, the study identified a concerning prevalence of polypharmacy in the study population. Unsurprisingly, polypharmacy has been known to be related to adverse outcomes in dementia populations, including unplanned hospitalization [16]. This highlights the potential risks associated with drug interactions and adverse effects. Second, the study demonstrated a high rate of readmission within 30 days, emphasizing the necessity for enhanced transitional care and follow-up for older adults with dementia following hospitalization. To date, no successful interventions have been identified that effectively reduce the number of hospital admissions among individuals with dementia who reside in the community [17]. Further studies would be beneficial to explore strategies aimed at reducing readmissions among this population.

The median survival time in our cohort was 3.06 years after hospitalization. When comparing our results to a previous prospective cohort study conducted in a large urban general hospital in North London [8], we observed that our study population had a longer median survival time. This discrepancy can potentially be attributed to differences in the methodology employed for data collection. In our study, we relied on the existing diagnoses of dementia documented in the medical records, whereas the previous study in London actively screened for all cases within the admission. In addition, it is worth noting that our study population included relatively younger individuals, and we excluded cases of individuals who passed away during their hospital stay. Also, we did not exclude cases with brief hospitalization, which often indicates less severe conditions. These factors potentially contributed to the overall longer survival observed in our study.

Our findings suggest that there are multiple factors that may contribute to increased mortality risk in older adults with dementia who survived hospitalization. The correlation between age and mortality that our study revealed is certainly associated with the age-specific mortality rate within an overall population. Decline in cognition, function, and physiology are signs of aging. These changes often result in increased susceptibility to illness and disability. Consistent with previous studies [18–20], this finding indicates that advancing age is significantly correlated with increased mortality rates in individuals with dementia.

Additionally, our study identified an elevated mortality risk associated with a diagnosis of mixed-type dementia in comparison to Alzheimer’s disease. Management and treatment of mixed dementia pose significant challenges, and existing regimens of medication provide modest clinical benefits [21, 22]. This implies that mixed dementia may have a more severe clinical course than Alzheimer’s disease alone, which could contribute to the increased mortality risk. This is consistent with previous research, which has shown that individuals with mixed-type dementia have a median lower survival time than those with Alzheimer’s disease or vascular dementia alone [23]. However, another study found that mixed dementia had an intermediate hazard ratio between Alzheimer’s disease and vascular dementia, while frontotemporal dementia presented the highest risk [24].

The CCI score has been shown to be a strong predictor of mortality and other adverse outcomes in various populations [25], including those with dementia. The findings from our study which indicated a high CCI score as a significant predictor of mortality in people with dementia are consistent with the results of previous studies [26, 27], which also showed that these patients have a heavy comorbidity burden. These findings emphasize the importance of managing comorbidities in people with dementia as a crucial aspect of their care.

Pressure ulcers were found to significantly increase the risk of mortality, consistent with previous research indicating their prevalence in dementia patients [28, 29]. These ulcers can lead to infections, sepsis, and death [30]. Patients with advanced dementia and pressure ulcers had a much lower median survival rate than those without dementia [31, 32]. Patients with dementia faced a significantly higher risk of developing pressure ulcers [33]. The development of pressure ulcers is often considered an indicator of inadequate care [33], and the prevention and management of these wounds are crucial components of comprehensive dementia care.

High serum creatinine levels were found to be a significant predictor of mortality. Impaired kidney function, as reflected by high serum creatinine levels [34], can contribute to the development or worsening of comorbidities and of behavioral and psychological symptoms of dementia. Aging can also have impact on medication clearance and toxicity through alterations in pharmacokinetics and pharmacodynamics [35]. This can result in an elevated risk of adverse drug events among older patients, particularly those with impaired renal or liver function [36]. Inappropriately prescribed medications are frequently observed among older people with renal impairment or dementia [37], which can further increase the likelihood of adverse drug events and subsequent hospitalization. Consequently, identification and management of medication-related issues in older people with renal impairment and dementia are imperative to mitigate the risk of unfavorable outcomes.

Our study found that endotracheal intubation impacts mortality. A history of the insertion of an endotracheal tube during admission implies the severity of the disease related to hospitalization. Greater severity could lead to higher mortality. Moreover, elderly individuals demonstrate an increased vulnerability to airway complications due to the progressive deterioration of the airway, coupled with other pathophysiologies and cognitive changes [38]. Notably, advancing age was identified as an independent factor associated with a heightened risk of mortality [39]. The risk of tissue injury and hypoxemia is notably higher at extreme ages. Additionally, a greater incidence of vocal cord paralysis is observed in older individuals [40].

Finally, the rate of readmission within 30 days was 24.86%, a finding consistent with previous studies showing high rates of re-visits to the emergency department and readmissions in dementia patients [41–43]. Several clinical factors also predict hospital readmissions in dementia patients. The severity of dementia, uncontrolled comorbidities, and inadequate caregiver support could lead to readmissions [10, 44]. Recognition of these factors may assist healthcare providers in identifying high-risk patients and targeting readmission interventions. These findings emphasize the importance of comprehensive discharge planning and post-discharge follow-up for dementia patients to avoid hospital readmissions.

This study had several strengths. Firstly, it contributes to the existing body of evidence on post-admission mortality among older adult individuals with dementia, an area where current evidence is limited. By adding to this knowledge and endeavoring to fill the gap, the study enhances our understanding of the topic and provides valuable insights for healthcare professionals and researchers. Secondly, the meticulous collection of detailed information regarding the medical history of the participants means the findings can be accepted with confidence. This comprehensive approach enabled a thorough examination of the factors associated with mortality in older adult individuals with dementia in a hospital setting. This level of detail is crucial for further consideration of potential interventions that could positively impact patient outcomes among this population.

This study is not without its limitations. Firstly, we acknowledge that the number of dementia admissions over the 7 years in our study is surprisingly low. This could be attributed to several factors: First, the older population might be underdiagnosed for dementia. Second, the majority of Thai people have universal coverage, allowing access to healthcare through government hospitals, which are not the primary access points for our medical school. Consequently, they may not be diagnosed or treated at our institute. As a super tertiary hospital covering a small catchment area in northern Thailand, the cases we handle are mostly complicated. Third, our study is based on electronic medical records, which may result in underreporting. However, to address this issue, there is an auditing process in our hospital, making this less likely to occur. It should be noted that the study was retrospective, which means there is a possibility of missing data in the medical records. However, since the missing data was less than 10%, it is less likely that the results of subsequent statistical analyses were biased [45]. Secondly, while collecting the FAST (Functional Assessment Staging Test) for dementia would have been useful in estimating the disease severity in our study, we found that it was rarely noted in the records. Consequently, we decided to assess the severity of the disease using the Thai Mental Status Examination, as there is evidence to suggest that the MMSE (Mini-Mental State Examination) scores are related to FAST scores in estimating severity [46]. Thirdly, it is important to acknowledge that the study was conducted at a single center, which may limit the generalizability of the findings to other healthcare settings or populations. Replicating the study in multiple centers or different geographic locations would provide a more comprehensive understanding of the topic. Furthermore, factors associated with mortality may differ based on the nature of the population, when the ratio of deceased to living individuals could impact the results. Caution should be exercised when interpreting the findings in comparison to other study populations with different demographics than those in our study. Finally, it is worth mentioning that the study did not specifically aim to compare the study population with both the population without dementia and with dementia who had never been admitted. Consequently, the differences between these populations could not be explored within the scope of this study. To gain further insights, future studies should focus on investigating and providing detailed comparisons between the study population and these other specific populations.

## Conclusions

Our study identified several notable predictors of mortality, including older age, higher comorbidity, a higher serum creatinine level, insertion of endotracheal tube. and readmission within 30 days. These findings emphasize the urgent need for more effective and personalized management strategies in the hospital setting for these vulnerable patients. Knowing these factors help to inform and emphasize risk prediction for early initiation of palliative care and improve the care for patients at high risk of mortality, particularly addressing treatable or modifiable factors. Healthcare providers can use the findings to identify patients who may be at higher risk of mortality and develop targeted interventions which may improve patient outcomes. We are optimistic that our study will contribute to enhancing outcomes and improving the quality of care for this growing population.

### Electronic supplementary material

Below is the link to the electronic supplementary material.

Supplementary Material 1

## Data Availability

The datasets used and/or analyzed during the current study are available from the corresponding author on reasonable request.

## List of abbreviations

aHRs: Adjusted hazard ratios
BPSD: Behavioral and psychological symptoms of dementia
CCI: Charlson Comorbidity Index
CI: Confidence intervals
ICD: International Classification of Diseases
TMSE: Thai Mental State Examination
