# Supplementary material for: Survival analysis of older adults with dementia: predicting factors after unplanned hospitalization in Maharaj Nakorn Chiang Mai Hospital
Source: BMC Geriatr. 2024 Jan 3;24:11. doi: 10.1186/s12877-023-04558-x (PMC10765674; doi:10.1186/s12877-023-04558-x)
Supplement: Supplementary file 1 — Supplementary Material 1 [file 12877_2023_4558_MOESM1_ESM.docx]

**Supplementary Table 1** Diagnosis of Dementia Regarding ICD-10

| **Primary diagnosis by ICD-10** | **N(%)** |
| --- | --- |
| F00.0 Dementia in Alzheimer disease with early onset | 4 (2.21) |
| F00.1 Dementia in Alzheimer disease with late onset | 3 (1.66) |
| F00.2 Dementia in Alzheimer disease, atypical or mixed type | 1 (0.55) |
| F00.9 Dementia in Alzheimer disease, unspecified | 5 (2.76) |
| F01.3 Mixed cortical and subcortical vascular dementia | 1 (0.55) |
| F01.8 Other vascular dementia | 6 (3.31) |
| F01.9 Vascular dementia, unspecified | 56 (30.94) |
| F02.0 Dementia in Pick disease | 1 (0.55) |
| F02.3 Dementia in Parkinson disease | 23 (12.71) |
| F03 Unspecified dementia | 20 (11.05) |
| G30.1 Alzheimer disease with late onset | 22 (12.15) |
| G30.8 Other Alzheimer disease | 1 (0.55) |
| G30.9 Alzheimer disease, unspecified | 37 (20.44) |
| G31.9 Degenerative disease of nervous system, unspecified | 1 (0.55) |

**Supplementary Table 2** Primary diagnosis

| Primary diagnosis | N (%) |
| --- | --- |
| Pneumonia | 25 (13.81) |
| Neurodegenerative diseases | 18 (9.96) |
| Urinary tract infection | 13 (7.18) |
| Fractures and osteoporosis | 13 (7.18) |
| Cerebrovascular accident | 12 (6.64) |
| Coronary artery disease | 9 (4.98) |
| Intracranial haemorrhage | 6 (3.31) |
| Skin infection | 6 (3.31) |
| Neoplasms | 5 (2.76) |
| Gastrointestinal hemorrhage | 5 (2.76) |
| Heart failure | 5 (2.76) |
| Hydrocephalus | 5 (2.76) |
| Anemia or hematologic disorders | 4 (2.21) |
| Sepsis or septic shock | 4 (2.21) |
| Diabetic-related condition | 3 (1.66) |
| Delirium | 3 (1.66) |
| Seizure | 3 (1.66) |
| Bowel obstruction | 3 (1.66) |
| Renal failure | 3 (1.66) |
| Aortic disease | 3 (1.66) |
| Sleep apnoea | 2 (1.10) |
| Cataract | 2 (1.10) |
| Chronic obstructive pulmonary disease with acute exacerbation | 2 (1.10) |
| Electrolyte imbalance | 2 (1.10) |
| Dysphagia | 2 (1.10) |
| Alteration of consciousness | 2 (1.10) |
| Renal stone | 2 (1.10) |
| Gallbladder and bile duct disease | 2 (1.10) |
| Headache | 1 (0.55) |
| Malnutrition | 1 (0.55) |
| Dengue hemorrhagic fever | 1 (0.55) |
| Crystal arthropathies | 1 (0.55) |
| Meningitis | 1 (0.55) |
| Pulmonary embolism | 1 (0.55) |
| Atrial fibrillation and flutter | 1 (0.55) |
| Gastroenteritis and colitis | 1 (0.55) |
| Tuberculosis | 1 (0.55) |
| Atherosclerosis of arteries of extremities | 1 (0.55) |
| Chronic sphenoidal sinusitis | 1 (0.55) |
| Status asthmaticus | 1 (0.55) |
| Acute appendicitis | 1 (0.55) |
| Diverticular disease of large intestine with perforation and abscess | 1 (0.55) |
| Dizziness | 1 (0.55) |
| Hypovolaemic shock | 1 (0.55) |
| Polyp of corpus uteri | 1 (0.55) |
| Anaphylaxis | 1 (0.55) |
